# Supply of interventional cardiologists and the provision of lower-value Percutaneous Coronary Interventions (PCI)

**DOI:** 10.1371/journal.pone.0352150

**Published:** 2026-07-22

**Authors:** Adam Gaffney, Kelsey Chalmers, David U. Himmelstein, Steffie Woolhandler, Vikas Saini

**Affiliations:** 1 Harvard Medical School, Boston, Massachusetts, United States of America; 2 Department of Medicine, Cambridge Health Alliance, Cambridge, Massachusetts, United States of America; 3 Lown Institute, Needham, Massachusetts, United States of America; 4 Hunter College, City University of New York, New York, United States of America; CUNY School of Medicine: The City College of New York CUNY School of Medicine, UNITED STATES OF AMERICA

## Abstract

**Background:**

Percutaneous coronary interventions (PCI) can be lifesaving for patients with acute coronary syndromes but of lower value for patients with stable coronary artery disease (CAD). Previous studies suggest that larger provider supply drives higher healthcare utilization. Whether this is true for lower-value PCI is unknown.

**Objective:**

To examine the association between the regional supply of PCI-performing physicians and PCI use and lower-value use.

**Design:**

Cross-sectional.

**Setting:**

100% Medicare fee-for-service (FFS) claims and Medicare Advantage encounter data, linked to publicly available sources of population and hospital data.

**Participants:**

All Medicare FFS and MA beneficiaries undergoing PCI at US hospitals, 2019–2021.

**Measurements:**

Our exposure was the supply of PCI-performing physicians per million population in the hospital-referral-region (HRR). Our primary outcome was lower-value PCI provision defined as the share of all PCIs performed for stable CAD at the hospital-level, unadjusted and with multilevel linear regression adjustment for hospital and regional-population factors. We also examined both PCI use and lower-value use at the regional (HRR) level.

**Results:**

Our final dataset included 1,580 hospitals across 306 HRRs with 5,505 PCI-performing physicians. In our primary, hospital-level analysis, the lower-value PCI provision rate averaged 17.3% among hospitals located in HRRs in the lowest quintile of PCI provider density and 22.6% among hospitals in HRRs in the highest quintile. In our fully adjusted model, hospitals in the highest provider density quintile had a 5.77 percentage point higher lower-value provision rate than those in the lowest quintile (95% CI 3.70, 7.84; p < 0.01).

In our HRR-level analysis, both the total number of PCIs/beneficiary and lower-value PCIs/beneficiary rose with increasing regional PCI-provider density.

**Conclusions:**

Among Medicare beneficiaries, the regional supply of PCI providers correlates with PCI overall and lower-value use. These findings support a role for health planning efforts to align workforce supply with community health needs to constrain both costs and the provision of low-value care.

## Introduction

Over 600,000 percutaneous coronary interventions (PCI) are performed in the US annually.[1] These procedures can be lifesaving for patients suffering acute myocardial infarction (AMI) or unstable coronary syndromes, [2] but are lower-value when performed for patients with stable coronary artery disease (CAD). For instance, several randomized trials have found that for patients with stable CAD, PCI does not decrease mortality more than medications alone.[3,4] Moreover, although some trials (including ORBITA II) suggest that PCI improves symptoms, [3,5] the first ORBITA study found that for patients already on appropriate medications, PCI offered no better symptom relief than a sham (placebo) procedure.[6] Nevertheless, many stable CAD patients in the US undergo PCI, raising concern that this invasive and expensive procedure is sometimes overused.[7,8]

Some studies suggest that more PCIs (for any indication) are performed in locales with a greater density of cardiologists and PCI facilities.[9–11] Those analyses are in keeping with a large body of evidence [12–17] — dating to Wennberg and Gittelsohn’s seminal 1973 study of regional variation in the supply and utilization of medical services in Vermont [18] — that have documented the “dominating importance of supply characteristics in explaining between-market variations.” [19] However, for PCI (as for many other medical services), it remains unclear whether an abundance of physicians and medical facilities increases overuse (i.e., the delivery of lower-value care), appropriate use (i.e., delivery of higher-value care), or both.

Similar to PCIs, c-sections are sometimes lifesaving, but high c-section rates have long been used as an indicator of potential overuse.[20–22] The share of all PCIs performed on patients with stable CAD has similarly been used as a metric to compare rates of potential overuse across hospitals.[7,8] For the present study, we used that metric to examine whether “lower-value PCIs” are more frequently performed in regions with more doctors who perform that procedure.

## Methods

### Data sources

We obtained data on the geographic supply of PCI-performing physicians (hereinafter “PCI supply”) from the Centers for Medicare & Medicaid Services (CMS).[23,24]

To identify the number of PCIs performed at each U.S short-term general or critical access hospital we analyzed 100% samples of 2019–2021 Medicare Fee-for-Service (FFS) and Medicare Advantage (MA) inpatient, outpatient, and carrier claims and encounter data, following previously described methods.[7,25] We excluded federal and specialty hospitals, and those in US territories, lacking emergency services, that had fewer than 50 admissions/year, or did not provide surgical procedures, leaving n = 3,926 hospitals. We also excluded hospitals performing fewer than 10 total or 10 low-value PCIs on Medicare beneficiaries over three years, leaving n = 1,580 hospitals.

Hospital-level characteristics were drawn from CMS’ Care Compare 2022, the American Hospital Association Annual Survey, US Census Bureau data, and RAND Corporation’s version of CMS’ Hospital Cost Reporting Information System (HCRIS) (2021 calendar year, August 2023 version).[26] Hospital market data was drawn from the Urban Institute.[27]

We assessed the total number of Medicare A + B beneficiaries, Medicare Advantage penetration, and characteristics of Medicare FFS beneficiaries in each region using CMS’ 2021 “Medicare Geographic Variation” public use file, which provides data at the Hospital Referral Region (HRR) level.[28] HRRs (which we also refer to as “regions”) are groupings of ZIP codes based on where most residents receive their tertiary care. State-level 2021 data on characteristics of the MA population were drawn from the CMS’s Medicare Advantage (MA) Geographic Variation Public Use File.[29]

Data on overall population demographics of each HRR were analyzed using ZIP-Code Tabulation Area (ZCTA)-level 2015–2019 American Community Survey (ACS) data (obtained from IPUMS), [30] while ZCTA-level data on (modelled) population chronic disease prevalence and health insurance coverage was obtained from the 2021 PLACES dataset.[31] We then aggregated all ZCTA-level data to the HRR level (S1 Appendix).

### Variables

#### Exposure.

Our primary exposure was HRR-level PCI provider density, i.e., the number of PCI-performing physicians/million total population, using an approach similar to a previous study.[11] Using the Medicare Physician & Other Practitioners by Provider and Service data’s n = 9,449,361 provider-service level records, we defined PCI providers as clinicians performing 11 + FFS PCIs in 2020 (S1 Appendix provides CPT/HCPCS codes).[32,33]

For sensitivity analyses, we defined these providers using two alternative approaches. First, we identified clinicians performing 11 + FFS cardiac catheterization procedures of any type (with or without intervention; S1 Appendix provides the CPT/HCPCS codes). Second, we identified physicians with a taxonomy of “interventional cardiologists” (regardless of procedures performed) together with “cardiologists” who performed at least 10 + FFS PCIs as defined in the main specification.

Finally, we summed the number of PCI/catheterization providers in each HRR using their ZIP code, [34] and calculated the PCI-provider density by dividing this sum by the HRR’s total population (calculated from the ACS). Each hospital was then assigned the PCI-provider density of its HRR.[34] Finally, we divided hospitals into PCI-provider density quintiles.

#### Outcomes.

Our primary outcome measure, adapted from previous studies, [7,8,25] was the hospital-level “lower-value PCI provision rate” defined as the proportion of total PCI procedures performed on Medicare beneficiaries with stable heart disease, i.e., with neither AMI nor unstable angina. To do so, we first identified all patients undergoing at least one intervention (coronary stenting or balloon angioplasty).

We categorized patients as having stable CAD if their diagnosis of ischemic heart disease or angina occurred at least 6 months prior to the procedure, and their PCI was *not* associated with an emergency department (ED) visit (which might indicate an AMI or unstable angina). For greater specificity, following previous work, [7] we additionally excluded from the lower-value group PCIs performed within 14 days of either any ED visit or any diagnostic-code entry for unstable angina/AMI.

For a secondary analysis of lower-value PCI provisions at the HRR level, we calculated the percent of PCI lower-value provision at the HRR-level by averaging the lower-value PCI share across hospitals within each HRR using weights equal to the total number of PCIs performed in each hospital during the study period. We additionally examined the number of total and lower-value PCIs per 1,000 Medicare beneficiaries at the HRR level (inclusive of lower-PCI-volume hospitals).

#### Covariates.

Several factors likely affect regional rates of PCI use, including: the overall burden of heart disease in the community; the demographic characteristics of the community’s overall and Medicare populations; and hospital characteristics.

Hence, we adjusted for potential confounding by assessing three sets of covariates: (A) characteristics of the overall population in the region, (B) characteristics of Medicare beneficiaries in the region, and (C) hospital-level factors.

Overall population factors included the percent of the adult population with coronary heart disease (CHD); population age (specified as 40–64, 65–74, and 75+); median household income; and the percent of the non-elderly adult population who were uninsured.

Medicare beneficiary characteristics (for FFS enrollees) included mean age, percent male, percent dual-eligible (i.e., Medicare-Medicaid enrolled), and the average Hierarchical Condition Category score — an indicator of average severity of illness — at the HRR level. Medicare beneficiary characteristics (for MA enrollees) included mean age, percent male, and percent dual eligible (at the state level). We also included an indicator for MA penetration at the HRR level.

Finally, our hospital-level characteristics included: urban vs. rural location; safety-net hospital status (hospitals in the top 20 percent of dual-eligible [Medicare + Medicaid coverage] days among all hospitals in the Lown Institute Hospital Index); academic medical center status (per the American Hospital Association); bed number; for-profit ownership; total capital assets (calculated from 2021 HCRIS data); and county-level hospital market concentration in quartiles (from the Urban Institute).[27]

Among our final sample of n = 1,580 hospitals, n = 2 had missing data on bed number, n = 1 had missing data on ownership status, n = 9 had missing data on capital assets, n = 20 had missing data on CHD or uninsurance prevalence, and n = 1 had missing data on hospital market concentration; these were dropped in analyses that adjusted for those variables.

### Analysis

We conducted our primary analyses at the hospital-level, with the hospital-level lower-value PCI provision rate our outcome variable and HRR-level PCI provider supply quintile as the exposure. We first examined hospital characteristics stratified by PCI-provider-density quintile. Second, we calculated the mean rate of hospital-level lower-value PCI provision across hospitals in each PCI provider density quintile. Third, we performed multilevel mixed effects linear regression models (with random intercepts by HRR to account for potential hospital similarities within HRRs) to examine the association between regional PCI-provider density and the share of PCIs at each hospital that were lower value. For our main regression analysis (and for sensitivity analyses), we performed five models: 1) unadjusted; 2) adjusted only for hospital-level factors; 3) adjusted only for HRR-population factors; 4) adjusted only for regional Medicare enrollee characteristics; and 5) adjusted for all covariates.

We conducted seven sets of sensitivity analyses. We first assessed whether our findings were driven by small hospitals by repeating our analyses excluding hospitals with < 250 beds. Second, since rural hospitals are less likely to have PCI infrastructure, we repeated analyses after excluding them. Third, we repeated analyses using the broader definition of PCI provider (i.e., including providers performing *any* type of cardiac catheterization). Fourth, we repeated analyses using the modified-taxonomy-based definition (i.e., all providers identified as “interventional cardiologists” regardless of procedures performed, along with “cardiologists” who performed >10 PCI procedures). Fifth, we repeated primary analyses excluding calendar year 2020, given the marked pandemic-related disruptions in care delivery that year. Sixth, we repeated analyses using ordinary least squares (OLS) linear regressions with weights equal to total PCIs provided by each hospital, and standard errors clustered at the HRR. Seventh, we repeated analyses specifying PCI-provider density as a continuous (not categorical) variable.

Finally, we performed secondary analyses at the HRR-level, examining average PCI volumes (overall and lower-value) per Medicare beneficiary, as well as percent overuse, among HRRs divided into quintiles based on PCI-provider supply. We also performed a regression examining the association between the raw number of PCI providers and total PCIs in HRRs.

Analyses were performed with SAS Enterprise Guide version 7.15 HF9 or Stata/SE 18. Analyses of 100% Medicare claims data was performed under WCG Institutional Review Board (formerly New England IRB) Sponsor Protocol WO-6484, which waived requirement for informed consent in this secondary analysis of data absent direct identifiers; this data was analyzed March – September 2023. Other datasets used in this study are public and/or de-identified; analysis of these datasets is not classified as human subjects research according to the Cambridge Health Alliance Institutional Review Board and the Common Rule.

## Results

Our primary analytic dataset included 1,580 hospitals. S1 Table provides their characteristics, stratified by PCI-provider-density quintile. In high PCI-provider-density HRRs the regional populations were older, a higher proportion of persons had CAD, and median incomes were lower. However, the characteristics of Medicare beneficiaries in those HRRs were similar across provider-density quintiles, except for a higher share of FFS enrollees with Medicaid in the lowest density quartile. Most (88.5%) PCI-performing hospitals were urban, although rural hospitals were more common in the higher PCI-provider-density quintiles. Hospitals in higher PCI-provider-density quintiles also had lower total capital assets and were less likely to be safety-net hospitals or academic medical centers.

**Fig 1:**
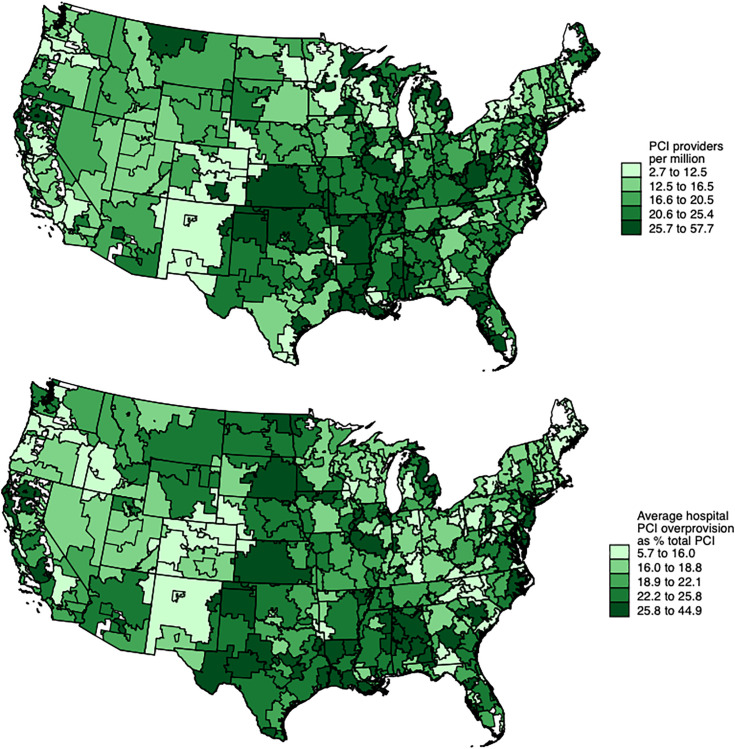
PCI Providers/Million Population (PCI-Provider Density) and Average Hospital Lower-Value PCI Provision Rate by Hospital Referral Region (HRR). Authors’ analysis of data on the geographic supply of PCI-performing clinicians from CMS, [23,24] and 100% sample of Medicare Fee-For-Service claims and Medicare Advantage encounter data from 2019-2021. HRR = Hospital Referral Region. Lower-value PCI provision rate = percent lower-value PCIs among total PCIs, calculated at the hospital level as described in the manuscript, and then averaged across all hospitals within each HRR, using weights equal to the total number of PCIs performed in each included hospital. For both PCI-provider density and lower-value PCI provision, HRRs are divided into quintiles of approximately equal numbers of HRRs for color-coded presentation in this map. We used shape files provided by the Dartmouth Atlas [35] (for HRRs) or IPUMS (for state/national borders) [30,36] to draw this map.

Fig 1 displays the geographic distribution of PCI-provider-density and the lower-value PCI provision rate averaged across hospitals within each HRR (weighted by total hospital PCI volume).

Provider density ranged from 2.7 to 57.7 providers per 1,000,000 population across HRRs (median = 18.9) and was highest in HRRs located in the Southern census region and lowest in the Western region (mean 22.5 vs. 15.4 per 1,000,000 population, respectively). Mean hospital lower-value PCI provision rates ranged from 5.7% to 44.9% across HRRs (median = 20.3%), and, like PCI-provider-density, was highest in HRRs in the South and lowest in the West.

**Fig 2:**
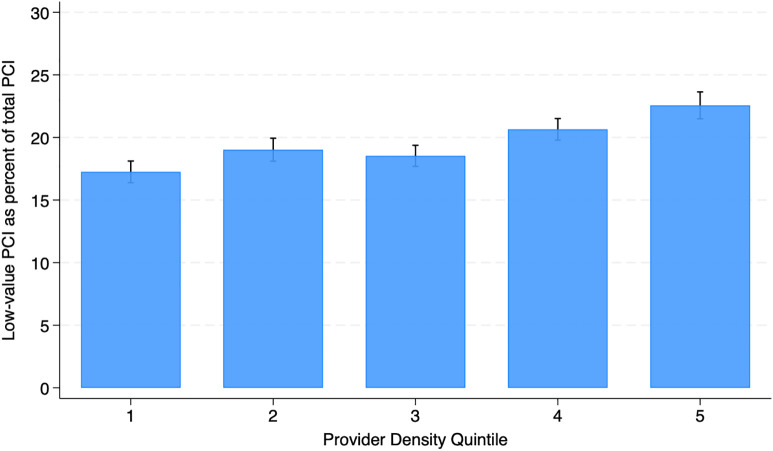
Mean Hospital Lower-Value PCI Provision Rate by HRR-level PCI-Provider Density Quintile (n = 1,580). Authors’ analysis of data on the geographic supply of PCI-performing clinicians from CMS, [23,24] and 100% sample of Medicare Fee-For-Service claims and Medicare Advantage encounter data from 2019–2021. PCI-provider density = PCI providers per 1,000,000 HRR population; hospitals were divided into quintiles by the PCI provider density, each with a roughly equal number of hospitals as follows: quintile 1 = 316 hospitals, quintile 2 = 317 hospitals, quintile 3 = 318 hospitals, quintile 4 = 315 hospitals, quintiles 5 = 314 hospitals.

Fig 2 provides the mean hospital lower-value PCI provision rate within PCI-provider-density quintiles.

**Fig 3:**
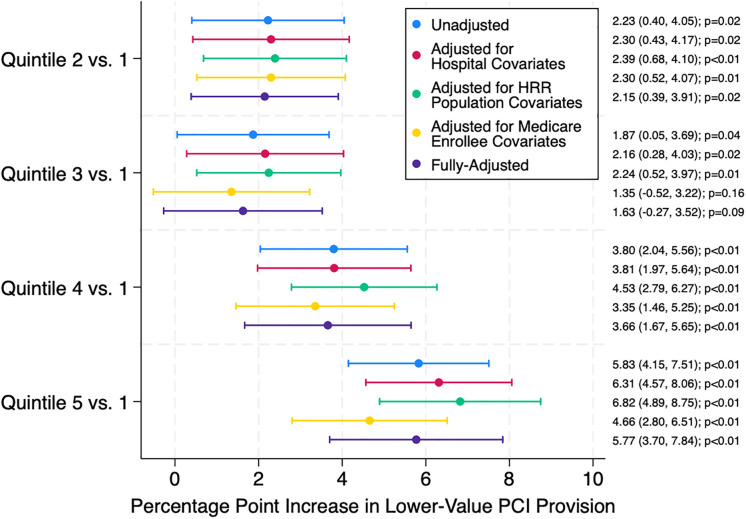
Unadjusted and Adjusted Association between the Hospital-Level Lower-Value PCI Provision Rate and HRR-level PCI-Provider Density Quintile (n = 1,580). Authors’ analysis of data on the geographic supply of PCI-performing clinicians from CMS [23,24] and 100% sample of Medicare Fee-For-Service claims and Medicare Advantage encounter data from 2019–2021, with covariate data drawn multiple data sources as described in the manuscript. PCI-Provider Density = PCI providers per 1,000,000 HRR population. Hospitals were divided into quintiles by the PCI provider density, each with a roughly equal number of hospitals as follows: quintile 1 = 316 hospitals, quintile 2 = 317 hospitals, quintile 3 = 318 hospitals, quintile 4 = 315 hospitals, quintiles 5 = 314 hospitals. See S1 Appendix for CPT codes used to define PCI providers. HRR Population covariates include the percentage of the HRR population aged 40–64 years, percent 65–74 years, and percent 75 + years; the percent of the non-elderly population that is uninsured; the percent of the adult population (18 years and older) with a physician diagnosis of coronary heart disease; and the median household income (inflation adjusted in 2021 dollars). Medicare Enrollee covariates include the average age, the percent male, the percent dual eligible, and the average Hierarchical Condition Code score (a measure of ill-health) of Medicare FFS beneficiaries residing in the HRR where the hospital is located, as well as indicator of Medicare Advantage penetration. Medicare Advantage enrollee characteristics include average age, percent male, and the percent dual eligible in the state in which the hospital is located. Hospital covariates include size (i.e., the number of hospital beds), safety-net hospital status (yes/no), urban/rural indicator, academic medical center status (yes/no), for-profit ownership (yes/no), and total hospital capital assets ($), as well as the quartile of hospital market concentration at the county level. Fully-adjusted models include all covariates. Regressions are Multilevel Linear Regressions that account for potential clustering within HRRs. Total hospitals in unadjusted analyses = 1,580; adjusted for hospital characteristics only = 1,570; adjusted for population characteristics only = 1,560; adjusted for Medicare FFS population characteristics only = 1,580; in fully adjusted analyses = 1,550.

The lower-value PCI provision rate rose from 17.3% in quintile 1 to 22.6% in quintile 5. Hospitals in quintiles 2–5 had significantly higher lower-value provision rates than those in quintile 1 in our unadjusted regressions (Fig 3).

Fig 3 also provides results from our multivariable regression models 2, 3, and 4, which adjust (individually) for HRR population characteristics, Medicare enrollee characteristics, hospital characteristics (respectively), and model 5, which adjusts for all characteristics. The differences we observed in unadjusted comparisons changed little with these adjustments. For example, compared to PCI-provider-density quintile 1, hospitals in quintile 5 had 5.83 percentage points higher lower-value PCI provision without adjustment (95% CI 4.15, 7.51; p < 0.01) and 5.77 percentage points higher provision in the fully adjusted model (95% CI 3.70, 7.84; p < 0.01).

Sensitivity analyses produced results broadly consistent with those in our principal analyses, including analyses restricted to urban hospitals (S1 Fig) or large hospitals (S2 Fig); that used a broader definition of PCI providers (S3 Fig) or a modified-taxonomy based definition (S4 Fig); that excluded calendar year 2020 procedures (S5 Fig); that employed PCI-weighted OLS regressions (S6 Fig); or that treated PCI-provider density as a continuous variable (S7 Fig).

S8 Fig provides results from our HRR-level secondary analyses, including total and lower-value PCIs per 1,000 Medicare enrollees, and average percent lower-value PCI provision in the HRR, again by PCI-provider-density quintile. From the lowest to highest provider-density quintile, mean total PCIs rose from 15.1 to 31.3, and lower-value PCIs from 2.6 to 7.9 per 1,000 Medicare beneficiaries. The average lower-value PCI provision rate also increased across quintiles. Each additional PCI provider in an HRR was associated with 225 additional PCIs (95% CI 218, 233; r-squared = 0.93).

## Discussion

In this nationwide analysis of Medicare FFS and Medicare Advantage beneficiaries, a greater supply of PCI-performing physicians was associated with higher rates of PCI total use and lower-value PCI provision. The correlation between PCI-provider-density and PCI utilization mirrors previous analyses that have found a link between the supply and use of medical services. However, our finding that a larger supply of resources is associated with a disproportionate increase in the provision of lower-value cardiac care is novel.

In a 1938 study, J. Alison Glover reported unexplained variations in tonsillectomy rates across British school districts, but did not examine whether this variation was explained by provider supply.[37] In the 1950s, Milton Roemer concluded that “hospital beds that are built tend to be used,” based on the observation that the availability of beds explained 70% of the variance in hospital use.[16] Studies from the 1960’s onwards, particularly those by John Wennberg and his Dartmouth colleagues, came to similar conclusions, finding (for instance) an association between regional hospital bed density and hospital use that was not explained by differences in population health.[12–14,18,38]

Several studies have specifically examined cardiac services. An analysis from northern New England found that the supply of cardiac-catheterization laboratories explained 85% of the variance in coronary angiography utilization, [10] a finding mirrored by a study finding a correlation between cardiologist supply and angiography rates in Ontario.[9] An analysis restricted to Medicare FFS beneficiaries also examined the relationship between PCI-provider supply and PCI utilization, but did not assess provision of lower-value PCI.[11] Our finding that PCI-provider supply explained approximately 90% of the regional variance in total PCI provision is consistent with these past observations.

The correlation between supply and total use that we observed could reflect appropriate supply in regions with greater need, underuse in low-supply areas, overuse in high-supply areas, or (most likely) a combination of these factors. For instance, we found that PCI-provider-density bore some relationship to population health needs, e.g., as measured by CAD prevalence. However, our finding that lower-value use increased more than total use with an increasing supply of PCI providers also implicates greater supply as a driver of potential overuse.

Few previous studies have examined the association between supply and low-value care utilization. One analysis found that Swiss dentists with more appointments available — a marker of over-supply — were more likely to offer unnecessary dental surgery.[39] Zhou et al. identified an association between overall bed and specialist supply and overuse of a basket of services [40] as measured by the Johns Hopkins Overuse Index (JHOI), a claims-based index based on the provision of 20 low-value procedures.[41] Similarly, using the JHOI, Segal et al. found that larger primary care supply was associated with less overuse, and investor ownership with more.[42] Our findings extend Zhou and colleagues’ findings by examining lower-value use as a share of total use for a specific, high cost procedure and the supply of specific proceduralists who deliver it.

John Wennberg and colleagues characterized supply-sensitive care as a driver of variation that is “unwarranted” [43] by the underlying prevalence of disease. “Unwarranted variation” has served as an indirect proxy for overuse, though it is imperfect because it assumes (among other things) that the “warranted” component of variation (i.e., due to differing population health needs) can be measured with sufficient precision to be fully adjusted away. To our knowledge, our study is the first to correlate a specific and direct measure of low-value use to resource supply without requiring assumptions about the “right” level of total PCI uptake in a population, reducing risk of bias from residual confounding. Our indicator of supply was indexed to (regional) population; future studies might examine how hospital-system level supply factors affect overuse.

Our study has several limitations. First, we relied on data for Medicare beneficiaries, and hence our findings may not be generalizable to patients with other forms of coverage. However, previous studies suggest that the relationship between regional supply and utilization is not payer-specific; [44] moreover, our inclusion of data on both Medicare FFS and Medicare Advantage beneficiaries improves on some previous studies.[11] Relatedly, our supply indicator was based on Medicare FFS provider data, although these include 99% of non-pediatric US physicians.[45] Two of our healthcare and health (including CHD prevalence) covariates were model-based rather than directly observed, although the PLACES data has been validated in comparisons to direct survey measures; [46] a claims-based indicator of CHD prevalence, moreover, would likely be endogenous to a PCI-based outcome.

Similar to many previous analyses, [7,8,40–42,47] we used Medicare claims data to measure potential overuse. Claims-based measures have advantages and disadvantages in the identification of low-value care: they permit identification of specific services prone to overuse, provide greater clinical detail than other hospital administrative records (but less detail than direct review of clinical records), and use already-collected data for the total Medicare-enrolled population.[8] While our metric of lower-value PCI provision used a claims-based method employed in previous studies, like other claims-based measures, this measure lacks the detailed clinical data (e.g., coronary anatomy, history of prior cardiac interventions, diagnostic testing results) needed to fully assess PCI-appropriateness for an individual patient. However, such claims-based metrics can be informative regarding the provision of low-value care at the population level. Moreover, while our claims-based measure of PCI-provider density would be expected to correlate with total PCI utilization, it would not be expected to correlate with low-value share. Finally, as with all observational studies, ours cannot prove causation.

Our findings regarding the association of supply and low-value use have policy implications. Some have advocated supply expansion as a tool to enhance competition and thereby lower health spending.[48,49] Our results suggest, however, that supply expansion may also have downsides, e.g., increased provision of low-value (as well as needed) services. Our findings also have implications for efforts to address the needs of underserved communities: current, market-guided incentives often lead hospitals to open new invasive cardiac programs near competing hospitals that already offer such services, increasing the risk of overuse rather than improving access.[50] Policies that target infrastructure expansion to underserved communities, rather than relying on revenue incentives, may be preferable. More broadly, our, and previous findings, [17] suggest that health planning might be one policy lever to reduce the provision of low-value care, contain costs and protect patients from unnecessary procedures — an important area for future research.

Before 1980, policy scholars — and some federal policies — focused on a health planning [51] approach to determine and implement the appropriate supply and distribution of expensive medical resources. Subsequently, planning gave way to reliance on market forces, payment incentives and managed care restrictions to contain costs and protect patients from unnecessary procedures. Our, and previous findings, [17] – and sharp cost increases in recent decades – suggest that health planning might be a more effective policy lever. Reinvigorated health planning could help target vital medical resources (such as PCI providers) to regions of need, and minimize redundancy, waste, and even harm to patients from low-value care provision.

## Supporting information

S1 Table Characteristics of the Regional General Population, Regional Medicare Population, and of Hospitals by HRR PCI Provider Density Quartile (n = 1,580 hospitals). Note: PCI = provider density = PCI providers per 1,000,000 HRR population; hospitals are divided into quintiles, each with a roughly equal number of hospitals. CHD Prevalence is among non-elderly adults. HCC = hierarchical condition category. Total capital assets in hundreds of millions of dollars. Of n = 1,580 hospitals, n = 20 with missing data on uninsurance or coronary heart disease prevalence; n = 2 with missing data on beds; n = 1 with missing data on ownership status; n = 9 with missing data on capital assets; n = 1 with missing data on hospital market concentration.
(DOCX)

S1 Appendix Additional Data on Methods. (DOCX)

S1 Fig Association between Hospital-Level Lower-Value PCI Provision Rate by HRR-level PCI Provider Density Quintile: Multivariable Multilevel Linear Regression Results, Urban Hospitals Only (n = 1,399). Note: PCI Provider Density is defined by PCI providers per 1,000,000 HRR population, with hospitals divided into quintiles (each with a roughly equal number of hospitals). See S1 Appendix for CPT codes used to define PCI providers. HRR population covariates include the percentage of the HRR population aged 40–64 years, percent 65–74 years, and percent 75 + years; the percent of the non-elderly population that is uninsured; the percent of the adult population (18 years and older) with a physician diagnosis of coronary heart disease; and the median household income (inflation adjusted in 2021 dollars). Medicare enrollee covariates include the average age, the percent male, the percent dual eligible, and the average Hierarchical Condition Code score (a measure of ill-health) of Medicare FFS beneficiaries residing in the HRR where the hospital is located, as well as indicator of Medicare Advantage penetration. Medicare Advantage enrollee characteristics include average age, percent male, and the percent dual eligible in the state in which the hospital is located. Hospital covariates include size (i.e., the number of hospital beds), safety-net hospital status (yes/no), urban/rural indicator, academic medical center status (yes/no), for-profit ownership (yes/no), and total hospital capital assets ($), as well as the quartile of hospital market concentration at the county level. Fully-adjusted models include all covariates. Regressions are Multilevel Linear Regressions that account for potential clustering within HRRs. Total hospitals in analyses that were unadjusted = 1,399; adjusted for hospital characteristics only = 1,390; adjusted for HRR population characteristics = 1,379; adjusted for Medicare FFS population characteristics = 1,399; in fully adjusted analyses = 1,370.
(TIF)

S2 Fig Association between Hospital-Level Lower-Value PCI Provision Rate by HRR-level PCI Provider Density Quintile: Multivariable Multilevel Linear Regression Results, Large Hospitals Only (n = 787). Note: PCI Provider Density is defined by PCI providers per 1,000,000 HRR population, with hospitals divided into quintiles (each with a roughly equal number of hospitals). See S1 Appendix for CPT codes used to define PCI providers. HRR population covariates include the percentage of the HRR population aged 40–64 years, percent 65–74 years, and percent 75 + years; the percent of the non-elderly population that is uninsured; the percent of the adult population (18 years and older) with a physician diagnosis of coronary heart disease; and the median household income (inflation adjusted in 2021 dollars). Medicare enrollee covariates include the average age, the percent male, the percent dual eligible, and the average Hierarchical Condition Code score (a measure of ill-health) of Medicare FFS beneficiaries residing in the HRR where the hospital is located, as well as indicator of Medicare Advantage penetration. Medicare Advantage enrollee characteristics include average age, percent male, and the percent dual eligible in the state in which the hospital is located. Hospital covariates include size (i.e., the number of hospital beds), safety-net hospital status (yes/no), urban/rural indicator, academic medical center status (yes/no), for-profit ownership (yes/no), and total hospital capital assets ($), as well as the quartile of hospital market concentration at the county level. Fully-adjusted models include all covariates. Regressions are Multilevel Linear Regressions that account for potential clustering within HRRs. Total hospitals in analyses that were unadjusted = 787; adjusted for hospital characteristics only = 782; adjusted for HRR population characteristics = 770; adjusted for Medicare FFS population characteristics = 787; in fully adjusted analyses = 765.
(TIF)

S3 Fig Association between Hospital-Level Lower-Value PCI Provision Rate by HRR-level Any Catheterization Provider Density Quintile: Multivariable Multilevel Linear Regression Results (n = 1,580). Note: PCI Provider Density = catheterization providers per 1,000,000 HRR population, with hospitals divided into quintiles (each with a roughly equal number of hospitals). See S1 Appendix for CPT codes used to define catheterization providers. HRR population covariates include the percentage of the HRR population aged 40–64 years, percent 65–74 years, and percent 75 + years; the percent of the non-elderly population that is uninsured; the percent of the adult population (18 years and older) with a physician diagnosis of coronary heart disease; and the median household income (inflation adjusted in 2021 dollars). Medicare enrollee covariates include the average age, the percent male, the percent dual eligible, and the average Hierarchical Condition Code score (a measure of ill-health) of Medicare FFS beneficiaries residing in the HRR where the hospital is located, as well as indicator of Medicare Advantage penetration. Medicare Advantage enrollee characteristics include average age, percent male, and the percent dual eligible in the state in which the hospital is located. Hospital covariates include size (i.e., the number of hospital beds), safety-net hospital status (yes/no), urban/rural indicator, academic medical center status (yes/no), for-profit ownership (yes/no), and total hospital capital assets ($), as well as the quartile of hospital market concentration at the county level. Fully-adjusted models include all covariates. Regressions are Multilevel Linear Regressions that account for potential clustering within HRRs. Total hospitals in analyses that were: unadjusted = 1,580; adjusted for hospital characteristics only = 1,570; adjusted for HRR population characteristics = 1,560; adjusted for Medicare FFS population characteristics = 1,580; fully adjusted analyses = 1,550.
(TIF)

S4 Fig Association between Hospital-Level Lower-Value PCI Provision Rate by HRR-level Taxonomy-Based Provider Density Quintile: Multivariable Multilevel Linear Regression Results (n = 1,580). Note: For this sensitivity analysis, as described in the text, providers were classified as PCI-providers if their taxonomy was listed as “Interventional Cardiologist” (regardless of procedures performed), or as “Cardiologist” (among those who performed PCI according to our primary specification). Provider Density = providers per 1,000,000 HRR population, with hospitals divided into quintiles (each with a roughly equal number of hospitals). See S1 Appendix for CPT codes used to define catheterization providers. HRR population covariates include the percentage of the HRR population aged 40–64 years, percent 65–74 years, and percent 75 + years; the percent of the non-elderly population that is uninsured; the percent of the adult population (18 years and older) with a physician diagnosis of coronary heart disease; and the median household income (inflation adjusted in 2021 dollars). Medicare enrollee covariates include the average age, the percent male, the percent dual eligible, and the average Hierarchical Condition Code score (a measure of ill-health) of Medicare FFS beneficiaries residing in the HRR where the hospital is located, as well as indicator of Medicare Advantage Penetration. Medicare Advantage enrollee characteristics include average age, percent male, and the percent dual eligible in the state in which the hospital is located. Hospital covariates include size (i.e., the number of hospital beds), safety-net hospital status (yes/no), urban/rural indicator, academic medical center status (yes/no), for-profit ownership (yes/no), and total hospital capital assets ($), as well as the quartile of hospital market concentration at the county level. Fully-adjusted models include all covariates. Regressions are Multilevel Linear Regressions that account for potential clustering within HRRs. Total hospitals in analyses that were: unadjusted = 1,580; adjusted for hospital characteristics only = 1,570; adjusted for HRR population characteristics = 1,560; adjusted for Medicare FFS population characteristics = 1,580; fully adjusted analyses = 1,550.
(TIF)

S5 Fig Unadjusted and Adjusted Association between the Hospital-Level Lower-Value PCI Provision Rate and HRR-level PCI-Provider Density Quintile Excluding Calendar Year 2020 procedures (n = 1,523). Authors’ analysis of data on the geographic supply of PCI-performing clinicians from CMS [23,24] and 100% sample of Medicare Fee-For-Service claims and Medicare Advantage encounter data from 2019 and 2021, with covariate data drawn multiple data sources as described in the manuscript. PCI-Provider Density = PCI providers per 1,000,000 HRR population. Hospitals were divided into quintiles by the PCI provider density, each with a roughly equal number of hospitals as follows: quintile 1 = 316 hospitals, quintile 2 = 317 hospitals, quintile 3 = 318 hospitals, quintile 4 = 315 hospitals, quintiles 5 = 314 hospitals. See S1 Appendix for CPT codes used to define PCI providers. HRR population covariates include the percentage of the HRR population aged 40–64 years, percent 65–74 years, and percent 75 + years; the percent of the non-elderly population that is uninsured; the percent of the adult population (18 years and older) with a physician diagnosis of coronary heart disease; and the median household income (inflation adjusted in 2021 dollars). Medicare enrollee covariates include the average age, the percent male, the percent dual eligible, and the average Hierarchical Condition Code score (a measure of ill-health) of Medicare FFS beneficiaries residing in the HRR where the hospital is located, as well as indicator of Medicare Advantage Penetration. Medicare Advantage enrollee characteristics include average age, percent male, and the percent dual eligible in the state in which the hospital is located. Hospital covariates include size (i.e., the number of hospital beds), safety-net hospital status (yes/no), urban/rural indicator, academic medical center status (yes/no), for-profit ownership (yes/no), and total hospital capital assets ($), as well as the quartile of hospital market concentration at the county level. Fully-adjusted models include all covariates. Regressions are Multilevel Linear Regressions that account for potential clustering within HRRs. Total hospitals in unadjusted analyses = 1,523; adjusted for hospital characteristics only = 1,514; adjusted for population characteristics only = 1,503; adjusted for Medicare FFS population characteristics only = 1,523; in fully adjusted analyses = 1,494.
(TIF)

S6 Fig Association between Hospital-Level Lower-Value PCI Provision Rate by HRR-level PCI-Provider Density Quintile: Ordinary Least Squares (OLS) Linear Regression Results (n = 1,580). Note: PCI-Provider Density =  PCI providers per 1,000,000 HRR population, with hospitals divided into quintiles (each with a roughly equal number of hospitals). See S1 Appendix for CPT codes used to define PCI providers. HRR population covariates include the percentage of the HRR population aged 40–64 years, percent 65–74 years, and percent 75 + years; the percent of the non-elderly population that is uninsured; the percent of the adult population (18 years and older) with a physician diagnosis of coronary heart disease; and the median household income (inflation adjusted in 2021 dollars). Medicare enrollee covariates include the average age, the percent male, the percent dual eligible, and the average Hierarchical Condition Code score (a measure of ill-health) of Medicare FFS beneficiaries residing in the HRR where the hospital is located, as well as indicator of Medicare Advantage penetration. Medicare Advantage enrollee characteristics include average age, percent male, and the percent dual eligible in the state in which the hospital is located. Hospital covariates include size (i.e., the number of hospital beds), safety-net hospital status (yes/no), urban/rural indicator, academic medical center status (yes/no), for-profit ownership (yes/no), and total hospital capital assets ($), as well as the quartile of hospital market concentration at the county level. Fully-adjusted models include all covariates. Regressions are ordinary least squares (OLS) linear regression models including weights equal to each hospitals’ total PCI volume, with standard errors clustered by HRR. Total hospitals in analyses that were unadjusted = 1,580; adjusted for hospital characteristics only = 1,570; adjusted for HRR population characteristics = 1,560; adjusted for Medicare FFS population characteristics = 1,580; in fully adjusted analyses = 1,550.
(TIF)

S7 Fig Association between Hospital-Level PCI Overprovision by HRR-level PCI Providers per Million: Multivariable Multilevel Linear Regression Results with PCI-Providers per Million Specified as a Continuous Variable (n = 1,580). Note: PCI Provider Density is defined by PCI providers per 1,000,000 HRR population. Percentage point increase in Lower-Value PCI Provision Rate is per a unit increase in providers per 1,000,000. See S1 Appendix for CPT codes used to define PCI providers. HRR population covariates include the percentage of the HRR population aged 40–64 years, percent 65–74 years, and percent 75 + years; the percent of the non-elderly population that is uninsured; the percent of the adult population (18 years and older) with a physician diagnosis of coronary heart disease; and the median household income (inflation adjusted in 2021 dollars). Medicare enrollee covariates include the average age, the percent male, the percent dual eligible, and the average Hierarchical Condition Code score (a measure of ill-health) of Medicare FFS beneficiaries residing in the HRR where the hospital is located, as well as indicator of Medicare Advantage Penetration. Medicare Advantage enrollee characteristics include average age, percent male, and the percent dual eligible in the state in which the hospital is located. Hospital covariates include size (i.e., the number of hospital beds), safety-net hospital status (yes/no), urban/rural indicator, academic medical center status (yes/no), for-profit ownership (yes/no), and total hospital capital assets ($), as well as the quartile of hospital market concentration at the county level. Fully-adjusted models include all covariates. Regressions are Multilevel Linear Regressions that account for potential clustering within HRRs. Total hospitals in analyses that were unadjusted = 1,580; adjusted for hospital characteristics only = 1,570; adjusted for HRR population characteristics = 1,560; adjusted for Medicare FFS population characteristics = 1,580; in fully adjusted analyses = 1,550.
(TIF)

S8 Fig PCI Utilization and Lower-Value PCI Provision Rate at the HRR Level (n = 306). Note: PCI-Provider Density = PCI providers per 1,000,000 population, with HRRs divided into quintiles with approximately equal numbers of HRRs by this indicator for analyses in this figure. PCI utilization is per 1,000 Medicare enrollees in the HRR. Note that for PCI utilization outcomes only (Panel A), we included an additional n = 235 hospitals with < 10 PCIs in the study period. The Lower-Value PCI Provision Rate rate (Panel B) is calculated as the weighted average of percent overuse among hospitals within each HRR, using weights equal to total PCIs provided by each hospital during the study period (n = 1,580).
(TIF)
