# Supplementary material for: Supply of interventional cardiologists and the provision of lower-value Percutaneous Coronary Interventions (PCI)
Source: PLoS One. 2026 Jul 22;21(7):e0352150. doi: 10.1371/journal.pone.0352150 (PMC13390938; doi:10.1371/journal.pone.0352150)
Supplement: S1 Table — Note: PCI = provider density = PCI providers per 1,000,000 HRR population; hospitals are divided into quintiles, each with a roughly equal number of hospitals. CHD Prevalence is among non-elderly adults. HCC = hierarchical condition category. Total capital assets in hundreds of millions of dollars. Of n = 1,580 hospitals, n = 20 with missing data on uninsurance or coronary heart disease prevalence; n = 2 with missing data on beds; n = 1 with missing data on ownership status; n = 9 with missing data on capital assets; n = 1 with missing data on hospital market concentration. (DOCX) [file pone.0352150.s001.docx]

**S1 Table: Characteristics of General and Medicare HRR Populations Hospitals by HRR PCI Provider Density Quartile (n=1,580)**

|  | PCI Provider Density Quintile | | | | |
| --- | --- | --- | --- | --- | --- |
|  | Quintile 1  n=316  (20.0%) | Quintile 2  n=317 (20.1%) | Quintile 3  n=318  (20.1%) | Quintile 4  n=315  (19.9%) | Quintile 5  n=314  (19.9%) |
| **General Population Characteristics: Mean (SD)** |  |  |  |  |  |
| Population 40-64 years (%) | 31.7 (2.0) | 31.9 (1.7) | 32.1 (1.4) | 32.0 (1.7) | 32.0 (1.6) |
| Population 65-74 years (%) | 8.5 (1.2) | 8.6 (1.4) | 9.1 (1.0) | 9.7 (1.2) | 10.4 (2.2) |
| Population 75+ years (%) | 6.1 (1.0) | 6.0 (1.4) | 6.6 (1.3) | 7.0 (1.0) | 7.8 (2.3) |
| CHD Prevalence (%) | 5.2 (0.9) | 5.6 (0.8) | 5.9 (0.8) | 6.3 (0.8) | 7.2 (0.9) |
| Uninsured (% Non-Elderly Adults) | 16.6 (7.3) | 17.8 (6.7) | 15.3 (4.2) | 16.3 (3.9) | 16.3 (4.4) |
| Median Income (Tens of Thousands $) | 7.3 (1.9) | 6.9 (1.2) | 6.9 (1.3) | 6.2 (1.1) | 5.5 (1.1) |
| **Medicare Population Characteristics: Mean (SD)** |  |  |  |  |  |
| FFS Beneficiaries Average Age (HRR) | 72.2 (1.2) | 72.3 (0.8) | 72.5 (1.0) | 72.4 (0.8) | 72.1 (1.1) |
| FFS Beneficiaries % Male (HRR) | 45.7 (1.4) | 45.5 (1.4) | 45.3 (1.3) | 45.4 (1.2) | 45.7 (1.4) |
| FFS Beneficiaries Average HCC Score (HRR) | 1.0 (0.1) | 1.0 (0.1) | 1.0 (0.1) | 1.0 (0.1) | 1.0 (0.1) |
| FFS Beneficiaries Dual Eligible (%) | 24.4 (9.9) | 16.3 (6.3) | 15.0 (4.0) | 13.7 (3.7) | 17.1 (5.6) |
| Medicare Advantage (MA) Penetration (%) | 53.7 (13.8) | 49.5 (10.6) | 44.4 (9.3) | 45.4 (10.1) | 44.4 (10.9) |
| MA Beneficiaries Average Age (State) | 73.1 (0.9) | 72.4 (0.9) | 72.3 (1.0) | 72.0 (1.0) | 71.7 (1.2) |
| MA Beneficiaries % Male (State) | 43.9 (1.1) | 43.4 (1.0) | 43.7 (0.9) | 43.7 (0.8) | 43.9 (0.8) |
| MA Beneficiaries % Medicaid Eligible (State) | 21.1 (6.7) | 22.4 (5.8) | 23.0 (5.8) | 22.4 (5.0) | 23.8 (6.1) |
| **Hospital Characteristics: Mean (SD) or Number (%)** |  |  |  |  |  |
| Hospital Beds | 322 (218) | 337 (273) | 305 (221) | 322 (288) | 279 (193) |
| Total capital assets | 5.9 (6.3) | 5.8 (7.7) | 5.1 (5.7) | 4.9 (6.4) | 3.8 (3.7) |
| Hospital Market Concentration (County Quartile) |  |  |  |  |  |
| 1 | 122 (38.6%) | 158 (50.0%) | 93 (29.2%) | 43 (13.7%) | 61 (19.4%) |
| 2 | 82 (25.9%) | 58 (18.4%) | 77 (24.2%) | 123 (39.0%) | 70 (22.3%) |
| 3 | 54 (17.1%) | 44 (13.9%) | 100 (31.4%) | 111 (35.2%) | 90 (28.7%) |
| 4 | 58 (18.4%) | 56 (17.7%) | 48 (15.1%) | 38 (12.1%) | 93 (29.6%) |
| Safety Net Hospital |  |  |  |  |  |
| Not a safety net hospital | 229 (72.5%) | 275 (86.8%) | 288 (90.6%) | 289 (91.7%) | 288 (91.7%) |
| Safety Net Hospital | 87 (27.5%) | 42 (13.2%) | 30 (9.4%) | 26 (8.3%) | 26 (8.3%) |
| Urban Hospital |  |  |  |  |  |
| Rural hospital | 13 (4.1%) | 28 (8.8%) | 35 (11.0%) | 44 (14.0%) | 61 (19.4%) |
| Urban Hospital | 303 (95.9%) | 289 (91.2%) | 283 (89.0%) | 271 (86.0%) | 253 (80.6%) |
| Academic Medical Center |  |  |  |  |  |
| Not an AMC | 265 (83.9%) | 271 (85.5%) | 278 (87.4%) | 277 (87.9%) | 291 (92.7%) |
| Academic Medical Center | 51 (16.1%) | 46 (14.5%) | 40 (12.6%) | 38 (12.1%) | 23 (7.3%) |
| For-Profit Ownership |  |  |  |  |  |
| Not for-profit | 255 (80.7%) | 247 (78.2%) | 266 (83.6%) | 247 (78.4%) | 246 (78.3%) |
| For-profit | 61 (19.3%) | 69 (21.8%) | 52 (16.4%) | 68 (21.6%) | 68 (21.7%) |

Note: PCI = provider density = PCI providers per 1,000,000 HRR population; hospitals are divided into quintiles, each with a roughly equal number of hospitals. CHD Prevalence is among non-elderly adults. HCC = hierarchical condition category. Total capital assets in hundreds of millions of dollars. Of n=1,580 hospitals, n=20 with missing data on uninsurance or coronary heart disease prevalence; n=2 with missing data on beds; n=1 with missing data on ownership status; n=9 with missing data on capital assets; n=1 with missing data on hospital market concentration.
