# Supplementary material for: Supply of interventional cardiologists and the provision of lower-value Percutaneous Coronary Interventions (PCI)
Source: PLoS One. 2026 Jul 22;21(7):e0352150. doi: 10.1371/journal.pone.0352150 (PMC13390938; doi:10.1371/journal.pone.0352150)
Supplement: S1 Appendix — (DOCX) [file pone.0352150.s002.docx]

# S1 Appendix

## ZIP Code, ZIP Code Tabulation Area (ZCTA), Hospital Referral Region (HRR) Linkage

We used the 2019 Dartmouth Atlas provided crosswalk to link ZIP codes to hospital referral regions (HRRs).^1^ This permitted assignment of providers and hospitals to HRRs. However, the IPUMS NHGIS American Community Survey (ACS) and the PLACES dataset were at the level of the ZIP Code Tabulation Area (ZCTA), described by the US Census Bureau as “generalized areal representations of the geographic extent and distribution of the point-based ZIP Codes…”^2^ To align ZCTAs with HRRs, we first used a ZCTA to ZIP code crosswalk (the 2019 UDS Mapper Crosswalk^3^) to link ZCTAs with ZIP codes, and then used the 2019 Dartmouth Crosswalk to link ZIP codes to HRRs, similar to previous studies.^4–6^

The 2019 Dartmouth crosswalk contains n=40,866 ZIP codes that link to n=32,968 unique ZCTAs in the UDS Mapper. Because ZCTAs can encompass more than one ZIP code, it is possible for a ZCTA to cross the boundary of an HRR. However, we observed that this was true of only 0.2% or n=76 ZCTAs (linked to n=177 ZIP codes), about half of which were described as belonging to a “large customer” or PO box. We dropped this small number of observations such that each remaining n=32,892 ZCTA uniquely linked to an HRR (n=306 HRRs).

Of these n=32,892 ZCTAs, 100% had a match in the ACS data, and 98% (n=32,322) had a match in the PLACES dataset.

When aggregating ACS and PLACES data to the HRR level, we dropped the small number of ZCTAs with zero population in the ACS, leaving n=32,556 ZCTA observations. The population encompassed by these remaining ZCTAs represented nearly the total US population (98.7%, or n=323,836,420 of n=327,998,752 in the 2015-19 ACS population). When calculating averages of median household income, the percent uninsured, and the percent with coronary artery disease across ZCTAs within each HRR, we excluded the small number of ZCTAs with missing data, and used weights equal to the total population of the ZCTA, similar to previous work.^6^ The population share of each age group was calculated by adding population totals for all available ZCTAs in the ACS for each HRR. Because the 2021 PLACES lacked data for many indicators for communities in New Jersey, as noted in the documentation, our final analytic dataset had missing data on uninsurance, CHD, and median income for hospitals located in New Jersey HRRs (n=4 HRRs).

## Stata Commands

We used Stata’s *spmap* package for drawing maps,^7^ shape files provided by the Dartmouth Atlas (for HRRs) or IPUMS (for state/national borders),^8^ the *coefplot* package for preparing other figures,^9^ and Stata’s *mixed* command for our multilevel regressions. As per Dartmouth Atlas terms of use, we acknowledge: “The data set forth at ‘Supplemental Data’ of publication/press release was obtained from Dartmouth Atlas Data website, which was funded by the Robert Wood Johnson Foundation, The Dartmouth Clinical and Translational Science Institute, under award number UL1TR001086 from the National Center for Advancing Translational Sciences (NCATS) of the National Institutes of Health (NIH), and in part, by the National Institute of Aging, under award number U01 AG046830.”^10^

## CPT Codes

1. **Cardiac Catheterization CPT/HCPCS Codes**

| **Code** | **Description** |
| --- | --- |
| 92978 | Endoluminl ivus oct c 1^st^ |
| 92979 | Endoluminl ivus oct c ea |
| 93451 | Right heart cath |
| 93452 | Left hrt cath w/ventrclgrphy |
| 93453 | R&l hrt cath w/ventriclgrphy |
| 93454 | Coronary artery angio s&i |
| 93455 | Coronary art/grft angio s&i |
| 93456 | R hrt coronary artery angio |
| 93457 | R hrt art/grft angio |
| 93458 | L hrt artery/ventricle angio |
| 93459 | L hrt art/grft angio |
| 93460 | R&l hrt art/ventricle angio |
| 93461 | R&l hrt art/ventricle angio |
| 93462 | L hrt cath trnsptl puncture |
| 93463 | Drug admin & hemodynmic meas |
| 93464 | Exercise w/hemodynamic meas |
| 93505 | Biopsy of heart lining |
| 93563 | Inject congenital card cath |
| 93564 | Inject hrt congntl art/grft |
| 93565 | Inject l ventr/atrial angio |
| 93566 | Inject r ventr/atrial angio |
| 93567 | Inject suprvlv aortography |
| 93568 | Inject pulm art hrt cath |
| 93571 | Heart flow reserve measure |
| 93572 | Heart flow reserve measure |
| 93593 | R hrt cath chd nml nt cnj |
| 93594 | R hrt cath chd abnl nt cnj |
| 93595 | L hrt cath chd nm/abn nt cnj |
| 93596 | R&l hrt cath chd nml nt cnj |
| 93597 | R&l hrt cath chd abnl nt cnj |
| 93598 | Car outp meas drg cath chd |
| G0269 | Occlusive device in vein art |

Source: Drawn verbatim from the Centers for Medicare and Medicaid Services.^11^

1. **Percutaneous Coronary Interventions (PCI) CPT Codes**

| **Code** | **Description** |
| --- | --- |
| 92920 | percutaneous transluminal coronary angioplasty; single major coronary artery or branch |
| 92921 | percutaneous transluminal coronary angioplasty; each additional branch of a major coronary artery (list separately in addition to code for primary procedure) |
| 92924 | percutaneous transluminal coronary atherectomy, with coronary angioplasty when performed; single major coronary artery or branch |
| 92925 | percutaneous transluminal coronary atherectomy, with coronary angioplasty when performed; each additional branch of a major coronary artery (list separately in addition to code for primary procedure) |
| 92928 | percutaneous transcatheter placement of intracoronary stent(s), with coronary angioplasty when performed; single major coronary artery or branch |
| 92929 | percutaneous transcatheter placement of intracoronary stent(s), with coronary angioplasty when performed; each additional branch of a major coronary artery (list separately in addition to code for primary procedure) |
| 92933 | percutaneous transluminal coronary atherectomy, with intracoronary stent, with coronary angioplasty when performed; single major coronary artery or branch |
| 92934 | percutaneous transluminal coronary atherectomy, with intracoronary stent, with coronary angioplasty when performed; each additional branch of a major coronary artery (list separately in addition to code for primary procedure) |
| 92937 | percutaneous transluminal revascularization of or through coronary artery bypass graft (internal mammary, free arterial, venous), any combination of intracoronary stent, atherectomy and angioplasty, including distal protection when performed; single vessel |
| 92938 | percutaneous transluminal revascularization of or through coronary artery bypass graft (internal mammary, free arterial, venous), any combination of intracoronary stent, atherectomy and angioplasty, including distal protection when performed; each additional branch subtended by the bypass graft (list separately in addition to code for primary procedure) |
| 92941 | percutaneous transluminal revascularization of acute total/subtotal occlusion during acute myocardial infarction, coronary artery or coronary artery bypass graft, any combination of intracoronary stent, atherectomy and angioplasty, including aspiration thrombectomy when performed, single vessel |
| 92943 | percutaneous transluminal revascularization of chronic total occlusion, coronary artery, coronary artery branch, or coronary artery bypass graft, any combination of intracoronary stent, atherectomy and angioplasty; single vessel |
| 92944 | percutaneous transluminal revascularization of chronic total occlusion, coronary artery, coronary artery branch, or coronary artery bypass graft, any combination of intracoronary stent, atherectomy and angioplasty; each additional coronary artery, coronary artery branch, or bypass graft (list separately in addition to code for primary procedure) |
| 92973 | percutaneous transluminal coronary thrombectomy mechanical (list separately in addition to code for primary procedure) |
| 92974 | transcatheter placement of radiation delivery device for subsequent coronary intravascular brachytherapy (list separately in addition to code for primary procedure) |
| 92975 | thrombolysis, coronary; by intracoronary infusion, including selective coronary angiography |
| 92978 | endoluminal imaging of coronary vessel or graft using intravascular ultrasound (ivus) or optical coherence tomography (oct) during diagnostic evaluation and/or therapeutic intervention including imaging supervision, interpretation and report; initial vessel (list separately in addition to code for primary procedure) |
| 92979 | endoluminal imaging of coronary vessel or graft using intravascular ultrasound (ivus) or optical coherence tomography (oct) during diagnostic evaluation and/or therapeutic intervention including imaging supervision, interpretation and report; each additional vessel (list separately in addition to code for primary procedure) |
| 93571 | intravascular doppler velocity and/or pressure derived coronary flow reserve measurement (coronary vessel or graft) during coronary angiography including pharmacologically induced stress; initial vessel (list separately in addition to code for primary procedure) |
| 93572 | intravascular doppler velocity and/or pressure derived coronary flow reserve measurement (coronary vessel or graft) during coronary angiography including pharmacologically induced stress; each additional vessel (list separately in addition to code for primary procedure) |
| C9600 | percutaneous transcatheter placement of drug eluting intracoronary stent(s), with coronary angioplasty when performed; single major coronary artery or branch |
| C9601 | percutaneous transcatheter placement of drug-eluting intracoronary stent(s), with coronary angioplasty when performed; each additional branch of a major coronary artery (list separately in addition to code for primary procedure) |
| C9602 | percutaneous transluminal coronary atherectomy, with drug eluting intracoronary stent, with coronary angioplasty when performed; single major coronary artery or branch |
| C9603 | percutaneous transluminal coronary atherectomy, with drug-eluting intracoronary stent, with coronary angioplasty when performed; each additional branch of a major coronary artery (list separately in addition to code for primary procedure) |
| C9604 | percutaneous transluminal revascularization of or through coronary artery bypass graft (internal mammary, free arterial, venous), any combination of drug-eluting intracoronary stent, atherectomy and angioplasty, including distal protection when performed; single vessel |
| C9605 | percutaneous transluminal revascularization of or through coronary artery bypass graft (internal mammary, free arterial, venous), any combination of drug-eluting intracoronary stent, atherectomy and angioplasty, including distal protection when performed; each additional branch subtended by the bypass graft (list separately in addition to code for primary procedure) |
| C9606 | percutaneous transluminal revascularization of acute total/subtotal occlusion during acute myocardial infarction, coronary artery or coronary artery bypass graft, any combination of drug-eluting intracoronary stent, atherectomy and angioplasty, including aspiration thrombectomy when performed, single vessel |
| C9607 | percutaneous transluminal revascularization of chronic total occlusion, coronary artery, coronary artery branch, or coronary artery bypass graft, any combination of drug-eluting intracoronary stent, atherectomy and angioplasty; single vessel |
| C9608 | percutaneous transluminal revascularization of chronic total occlusion, coronary artery, coronary artery branch, or coronary artery bypass graft, any combination of drug-eluting intracoronary stent, atherectomy and angioplasty; each additional coronary artery, coronary artery branch, or bypass graft (list separately in addition to code for primary procedure) |

Source: Drawn verbatim from the Centers for Medicare and Medicaid Services.^12^

# Appendix References

1. Supplemental Data. Dartmouth Atlas DATA. Accessed May 16, 2023. https://data.dartmouthatlas.org/supplemental/

2. Bureau UC. ZIP Code Tabulation Areas (ZCTAs). Census.gov. Accessed October 24, 2023. https://www.census.gov/programs-surveys/geography/guidance/geo-areas/zctas.html

3. ZIP Code to ZCTA Crosswalk – UDS Mapper. Accessed April 11, 2022. https://udsmapper.org/zip-code-to-zcta-crosswalk/

4. Lin SC, Hammond G, Esposito M, Majewski C, Foraker RE, Joynt Maddox KE. Segregated Patterns of Hospital Care Delivery and Health Outcomes. *JAMA Health Forum*. 2023;4(11):e234172. doi:10.1001/jamahealthforum.2023.4172

5. Zahnd WE, Hung P, Shi SK, et al. Availability of hospital-based cancer services before and after rural hospital closure, 2008-2017. *The Journal of Rural Health*. 2023;39(2):416-425. doi:10.1111/jrh.12716

6. Fleischman W, Agrawal S, King M, et al. Association between payments from manufacturers of pharmaceuticals to physicians and regional prescribing: cross sectional ecological study. *BMJ*. 2016;354:i4189. doi:10.1136/bmj.i4189

7. Pisati M. SPMAP: Stata module to visualize spatial data. *Statistical Software Components*. Published online January 18, 2018. Accessed February 14, 2023. https://ideas.repec.org//c/boc/bocode/s456812.html

8. IPUMS. National Historical Geographic Information System. Accessed May 24, 2023. https://www.nhgis.org/

9. Jann B. Plotting Regression Coefficients and other Estimates. *The Stata Journal*. 2014;14(4):708-737. doi:10.1177/1536867X1401400402

10. Terms of Use. Dartmouth Atlas DATA. Accessed March 20, 2026. https://data.dartmouthatlas.org/terms-of-use/

11. Article - Billing and Coding: Cardiac Catheterization and Coronary Angiography (A52850). Accessed May 16, 2023. https://www.cms.gov/medicare-coverage-database/view/article.aspx?articleid=52850&ver=38&

12. Article - Billing and Coding: Percutaneous Coronary Interventions (A57479). Accessed May 16, 2023. https://www.cms.gov/medicare-coverage-database/view/article.aspx?articleId=57479
