# Supplementary figures and images for: Supply of interventional cardiologists and the provision of lower-value Percutaneous Coronary Interventions (PCI)
Source: PLoS One. 2026 Jul 22;21(7):e0352150. doi: 10.1371/journal.pone.0352150 (PMC13390938; doi:10.1371/journal.pone.0352150)

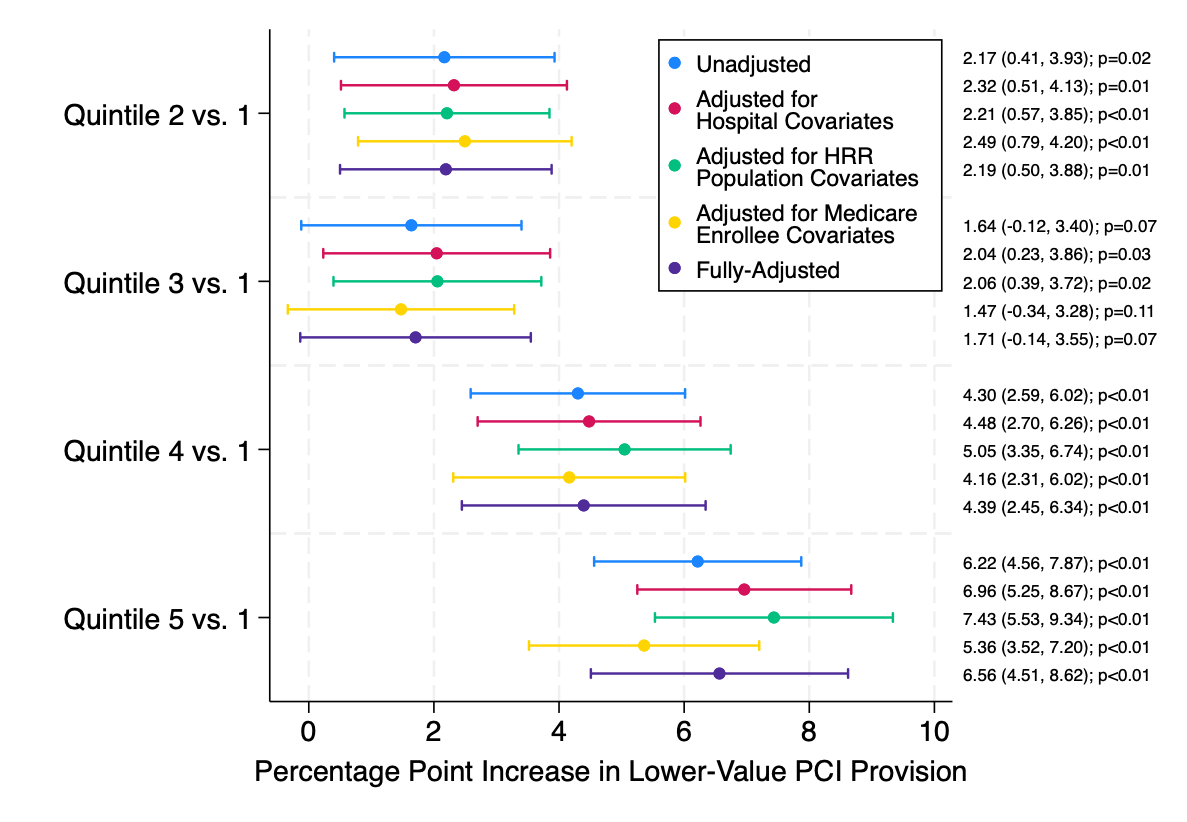

Supplement: S1 Fig — Note: PCI Provider Density is defined by PCI providers per 1,000,000 HRR population, with hospitals divided into quintiles (each with a roughly equal number of hospitals). See S1 Appendix for CPT codes used to define PCI providers. HRR population covariates include the percentage of the HRR population aged 40–64 years, percent 65–74 years, and percent 75 + years; the percent of the non-elderly population that is uninsured; the percent of the adult population (18 years and older) with a physician diagnosis of coronary heart disease; and the median household income (inflation adjusted in 2021 dollars). Medicare enrollee covariates include the average age, the percent male, the percent dual eligible, and the average Hierarchical Condition Code score (a measure of ill-health) of Medicare FFS beneficiaries residing in the HRR where the hospital is located, as well as indicator of Medicare Advantage penetration. Medicare Advantage enrollee characteristics include average age, percent male, and the percent dual eligible in the state in which the hospital is located. Hospital covariates include size (i.e., the number of hospital beds), safety-net hospital status (yes/no), urban/rural indicator, academic medical center status (yes/no), for-profit ownership (yes/no), and total hospital capital assets ($), as well as the quartile of hospital market concentration at the county level. Fully-adjusted models include all covariates. Regressions are Multilevel Linear Regressions that account for potential clustering within HRRs. Total hospitals in analyses that were unadjusted = 1,399; adjusted for hospital characteristics only = 1,390; adjusted for HRR population characteristics = 1,379; adjusted for Medicare FFS population characteristics = 1,399; in fully adjusted analyses = 1,370. (TIF) [file pone.0352150.s003.tif]

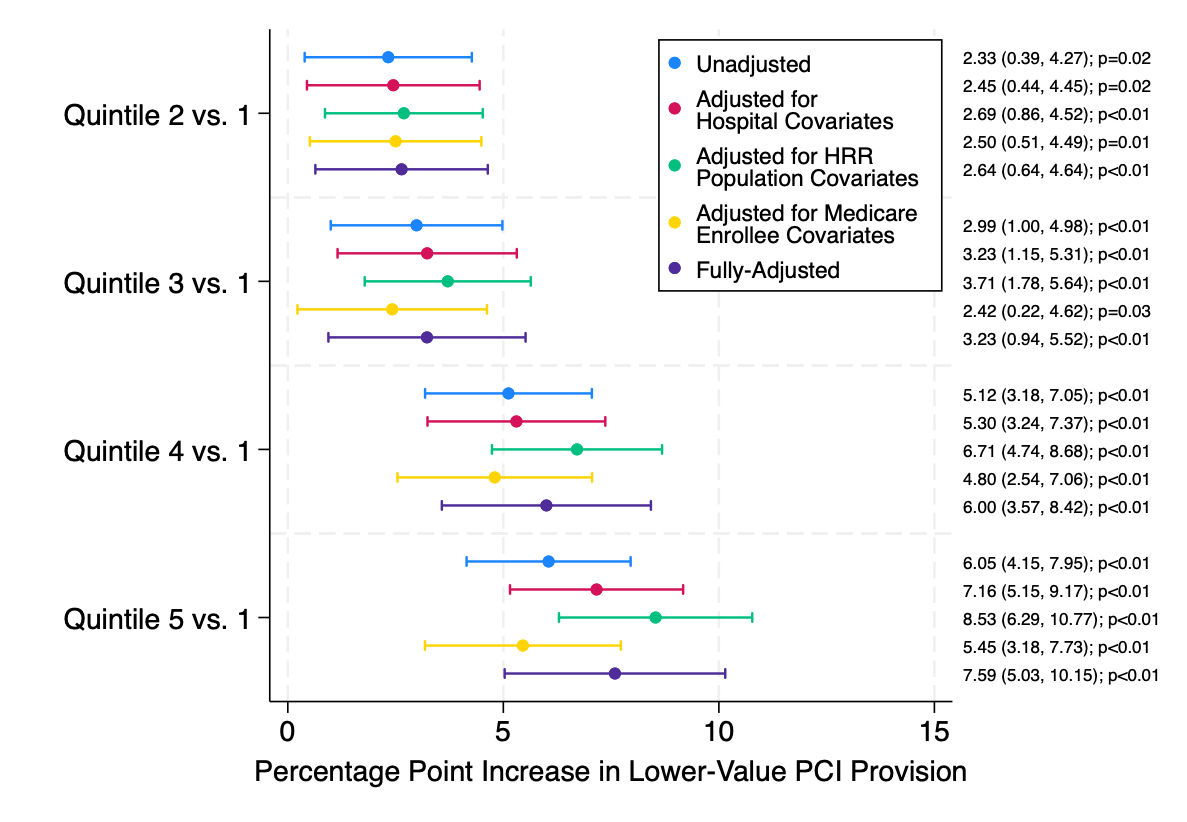

Supplement: S2 Fig — Note: PCI Provider Density is defined by PCI providers per 1,000,000 HRR population, with hospitals divided into quintiles (each with a roughly equal number of hospitals). See S1 Appendix for CPT codes used to define PCI providers. HRR population covariates include the percentage of the HRR population aged 40–64 years, percent 65–74 years, and percent 75 + years; the percent of the non-elderly population that is uninsured; the percent of the adult population (18 years and older) with a physician diagnosis of coronary heart disease; and the median household income (inflation adjusted in 2021 dollars). Medicare enrollee covariates include the average age, the percent male, the percent dual eligible, and the average Hierarchical Condition Code score (a measure of ill-health) of Medicare FFS beneficiaries residing in the HRR where the hospital is located, as well as indicator of Medicare Advantage penetration. Medicare Advantage enrollee characteristics include average age, percent male, and the percent dual eligible in the state in which the hospital is located. Hospital covariates include size (i.e., the number of hospital beds), safety-net hospital status (yes/no), urban/rural indicator, academic medical center status (yes/no), for-profit ownership (yes/no), and total hospital capital assets ($), as well as the quartile of hospital market concentration at the county level. Fully-adjusted models include all covariates. Regressions are Multilevel Linear Regressions that account for potential clustering within HRRs. Total hospitals in analyses that were unadjusted = 787; adjusted for hospital characteristics only = 782; adjusted for HRR population characteristics = 770; adjusted for Medicare FFS population characteristics = 787; in fully adjusted analyses = 765. (TIF) [file pone.0352150.s004.tif]

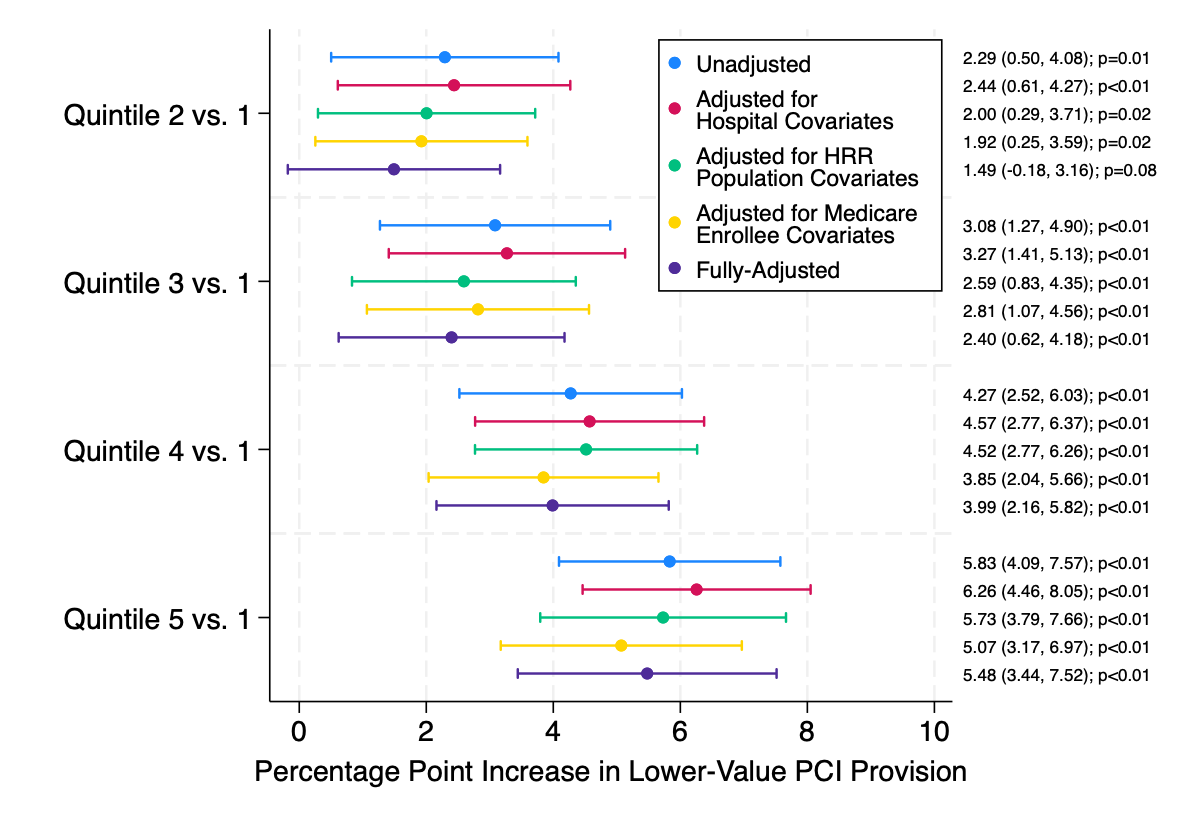

Supplement: S3 Fig — Note: PCI Provider Density = catheterization providers per 1,000,000 HRR population, with hospitals divided into quintiles (each with a roughly equal number of hospitals). See S1 Appendix for CPT codes used to define catheterization providers. HRR population covariates include the percentage of the HRR population aged 40–64 years, percent 65–74 years, and percent 75 + years; the percent of the non-elderly population that is uninsured; the percent of the adult population (18 years and older) with a physician diagnosis of coronary heart disease; and the median household income (inflation adjusted in 2021 dollars). Medicare enrollee covariates include the average age, the percent male, the percent dual eligible, and the average Hierarchical Condition Code score (a measure of ill-health) of Medicare FFS beneficiaries residing in the HRR where the hospital is located, as well as indicator of Medicare Advantage penetration. Medicare Advantage enrollee characteristics include average age, percent male, and the percent dual eligible in the state in which the hospital is located. Hospital covariates include size (i.e., the number of hospital beds), safety-net hospital status (yes/no), urban/rural indicator, academic medical center status (yes/no), for-profit ownership (yes/no), and total hospital capital assets ($), as well as the quartile of hospital market concentration at the county level. Fully-adjusted models include all covariates. Regressions are Multilevel Linear Regressions that account for potential clustering within HRRs. Total hospitals in analyses that were: unadjusted = 1,580; adjusted for hospital characteristics only = 1,570; adjusted for HRR population characteristics = 1,560; adjusted for Medicare FFS population characteristics = 1,580; fully adjusted analyses = 1,550. (TIF) [file pone.0352150.s005.tif]

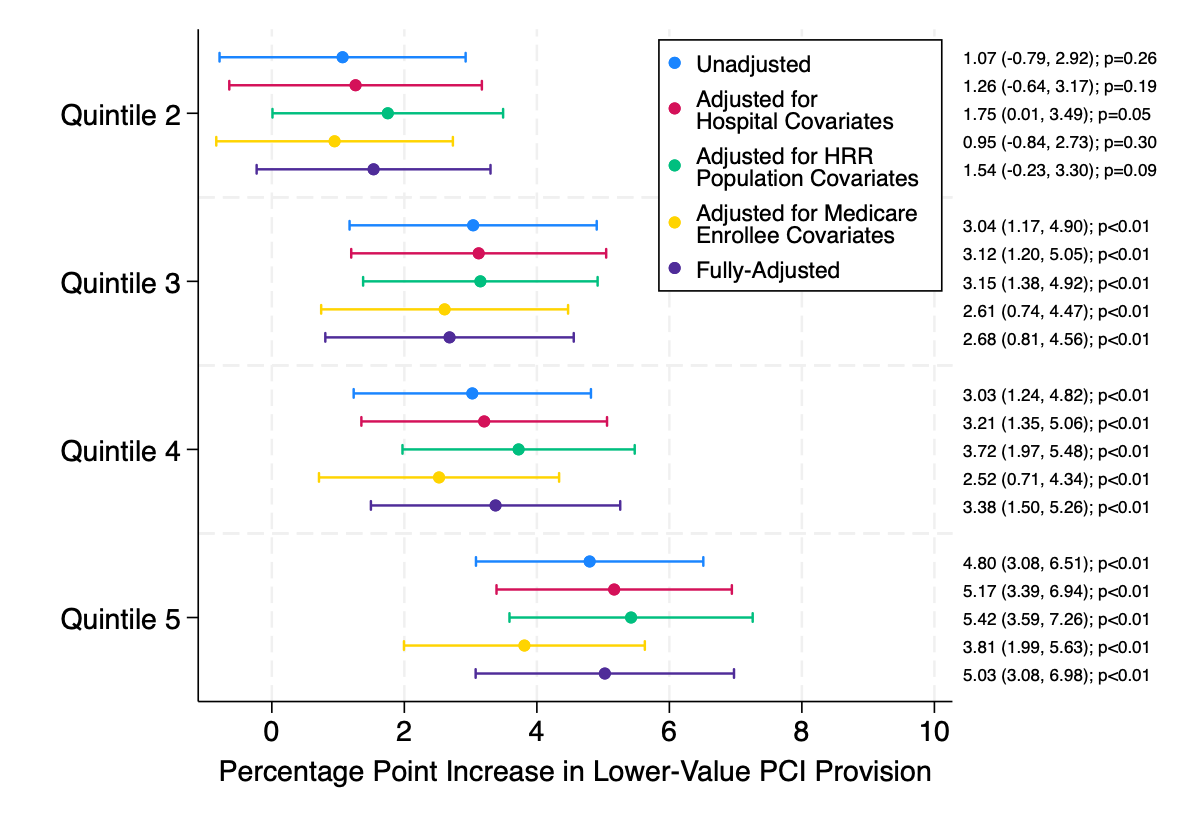

Supplement: S4 Fig — Note: For this sensitivity analysis, as described in the text, providers were classified as PCI-providers if their taxonomy was listed as “Interventional Cardiologist” (regardless of procedures performed), or as “Cardiologist” (among those who performed PCI according to our primary specification). Provider Density = providers per 1,000,000 HRR population, with hospitals divided into quintiles (each with a roughly equal number of hospitals). See S1 Appendix for CPT codes used to define catheterization providers. HRR population covariates include the percentage of the HRR population aged 40–64 years, percent 65–74 years, and percent 75 + years; the percent of the non-elderly population that is uninsured; the percent of the adult population (18 years and older) with a physician diagnosis of coronary heart disease; and the median household income (inflation adjusted in 2021 dollars). Medicare enrollee covariates include the average age, the percent male, the percent dual eligible, and the average Hierarchical Condition Code score (a measure of ill-health) of Medicare FFS beneficiaries residing in the HRR where the hospital is located, as well as indicator of Medicare Advantage Penetration. Medicare Advantage enrollee characteristics include average age, percent male, and the percent dual eligible in the state in which the hospital is located. Hospital covariates include size (i.e., the number of hospital beds), safety-net hospital status (yes/no), urban/rural indicator, academic medical center status (yes/no), for-profit ownership (yes/no), and total hospital capital assets ($), as well as the quartile of hospital market concentration at the county level. Fully-adjusted models include all covariates. Regressions are Multilevel Linear Regressions that account for potential clustering within HRRs. Total hospitals in analyses that were: unadjusted = 1,580; adjusted for hospital characteristics only = 1,570; adjusted for HRR population characteristics = 1,560; adjusted for [file pone.0352150.s006.tif]

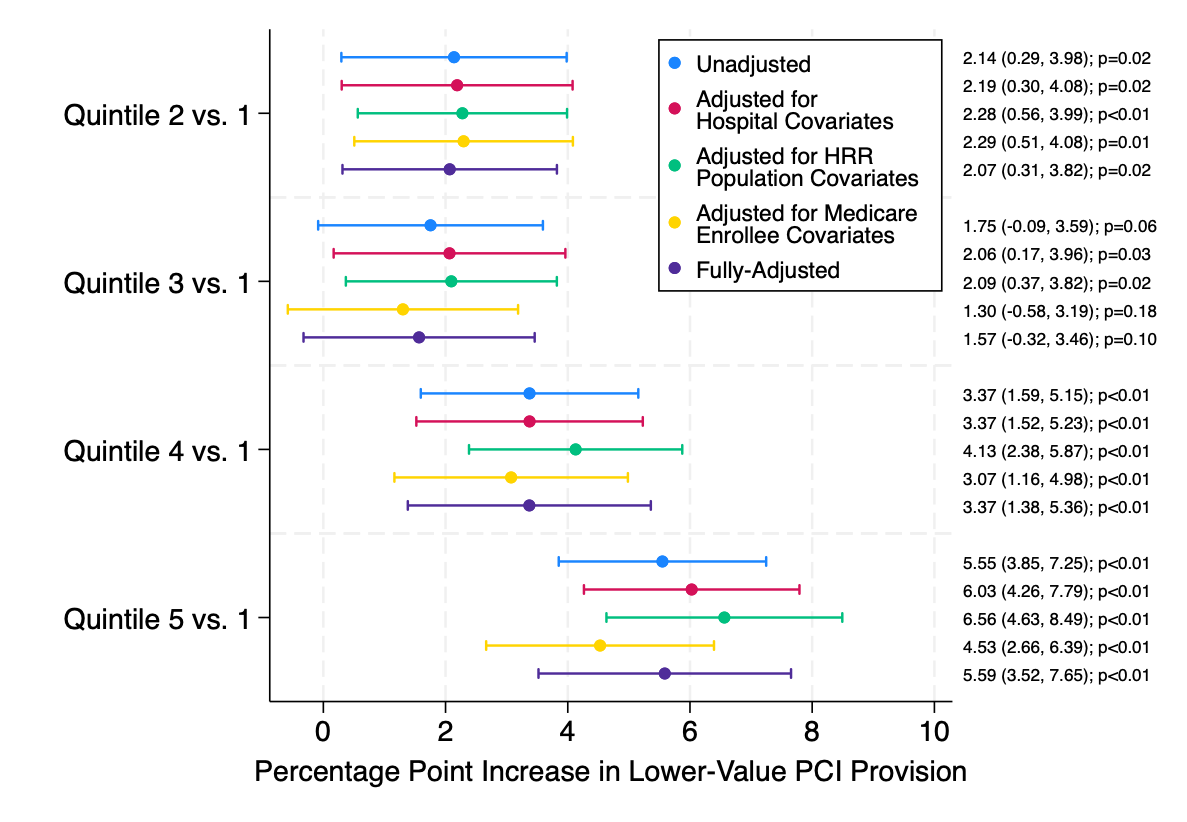

Supplement: S5 Fig — Authors’ analysis of data on the geographic supply of PCI-performing clinicians from CMS [23,24] and 100% sample of Medicare Fee-For-Service claims and Medicare Advantage encounter data from 2019 and 2021, with covariate data drawn multiple data sources as described in the manuscript. PCI-Provider Density = PCI providers per 1,000,000 HRR population. Hospitals were divided into quintiles by the PCI provider density, each with a roughly equal number of hospitals as follows: quintile 1 = 316 hospitals, quintile 2 = 317 hospitals, quintile 3 = 318 hospitals, quintile 4 = 315 hospitals, quintiles 5 = 314 hospitals. See S1 Appendix for CPT codes used to define PCI providers. HRR population covariates include the percentage of the HRR population aged 40–64 years, percent 65–74 years, and percent 75 + years; the percent of the non-elderly population that is uninsured; the percent of the adult population (18 years and older) with a physician diagnosis of coronary heart disease; and the median household income (inflation adjusted in 2021 dollars). Medicare enrollee covariates include the average age, the percent male, the percent dual eligible, and the average Hierarchical Condition Code score (a measure of ill-health) of Medicare FFS beneficiaries residing in the HRR where the hospital is located, as well as indicator of Medicare Advantage Penetration. Medicare Advantage enrollee characteristics include average age, percent male, and the percent dual eligible in the state in which the hospital is located. Hospital covariates include size (i.e., the number of hospital beds), safety-net hospital status (yes/no), urban/rural indicator, academic medical center status (yes/no), for-profit ownership (yes/no), and total hospital capital assets ($), as well as the quartile of hospital market concentration at the county level. Fully-adjusted models include all covariates. Regressions are Multilevel Linear Regressions that account for potential clustering within HRRs. Total hospitals [file pone.0352150.s007.tif]

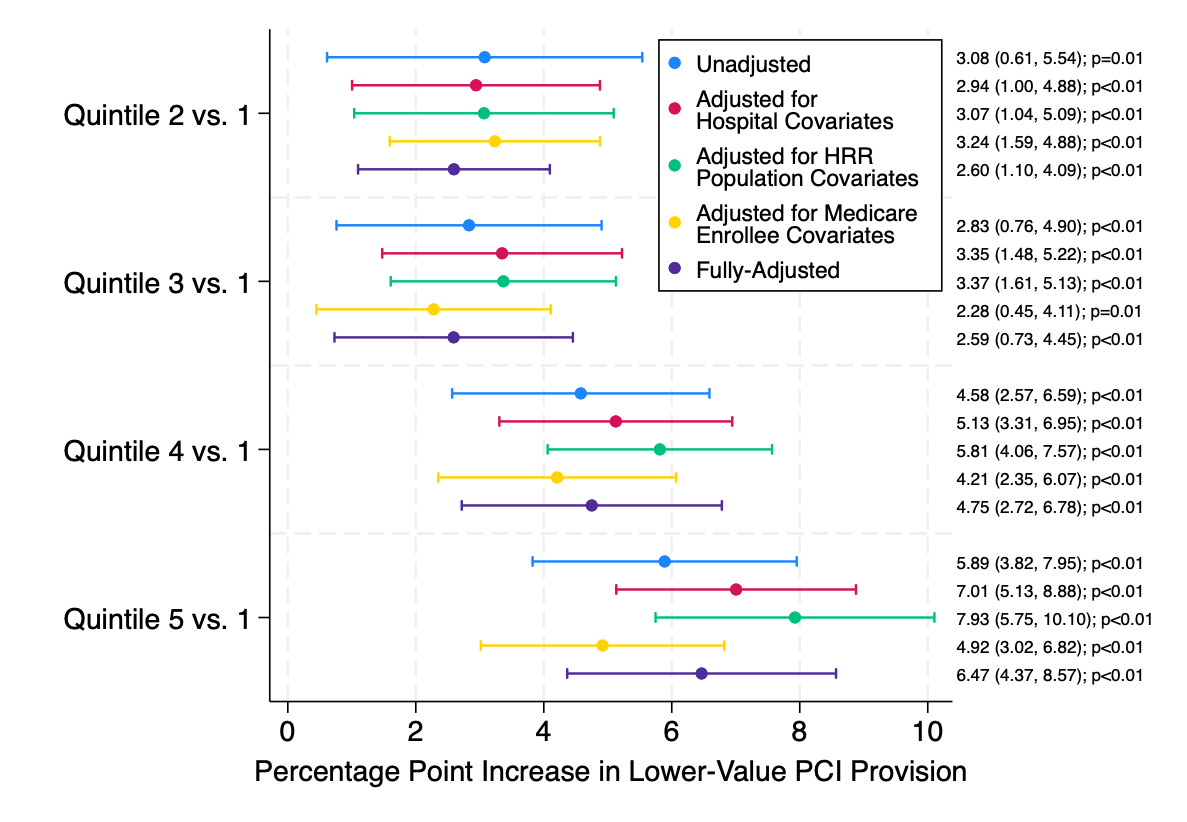

Supplement: S6 Fig — Note: PCI-Provider Density = PCI providers per 1,000,000 HRR population, with hospitals divided into quintiles (each with a roughly equal number of hospitals). See S1 Appendix for CPT codes used to define PCI providers. HRR population covariates include the percentage of the HRR population aged 40–64 years, percent 65–74 years, and percent 75 + years; the percent of the non-elderly population that is uninsured; the percent of the adult population (18 years and older) with a physician diagnosis of coronary heart disease; and the median household income (inflation adjusted in 2021 dollars). Medicare enrollee covariates include the average age, the percent male, the percent dual eligible, and the average Hierarchical Condition Code score (a measure of ill-health) of Medicare FFS beneficiaries residing in the HRR where the hospital is located, as well as indicator of Medicare Advantage penetration. Medicare Advantage enrollee characteristics include average age, percent male, and the percent dual eligible in the state in which the hospital is located. Hospital covariates include size (i.e., the number of hospital beds), safety-net hospital status (yes/no), urban/rural indicator, academic medical center status (yes/no), for-profit ownership (yes/no), and total hospital capital assets ($), as well as the quartile of hospital market concentration at the county level. Fully-adjusted models include all covariates. Regressions are ordinary least squares (OLS) linear regression models including weights equal to each hospitals’ total PCI volume, with standard errors clustered by HRR. Total hospitals in analyses that were unadjusted = 1,580; adjusted for hospital characteristics only = 1,570; adjusted for HRR population characteristics = 1,560; adjusted for Medicare FFS population characteristics = 1,580; in fully adjusted analyses = 1,550. (TIF) [file pone.0352150.s008.tif]

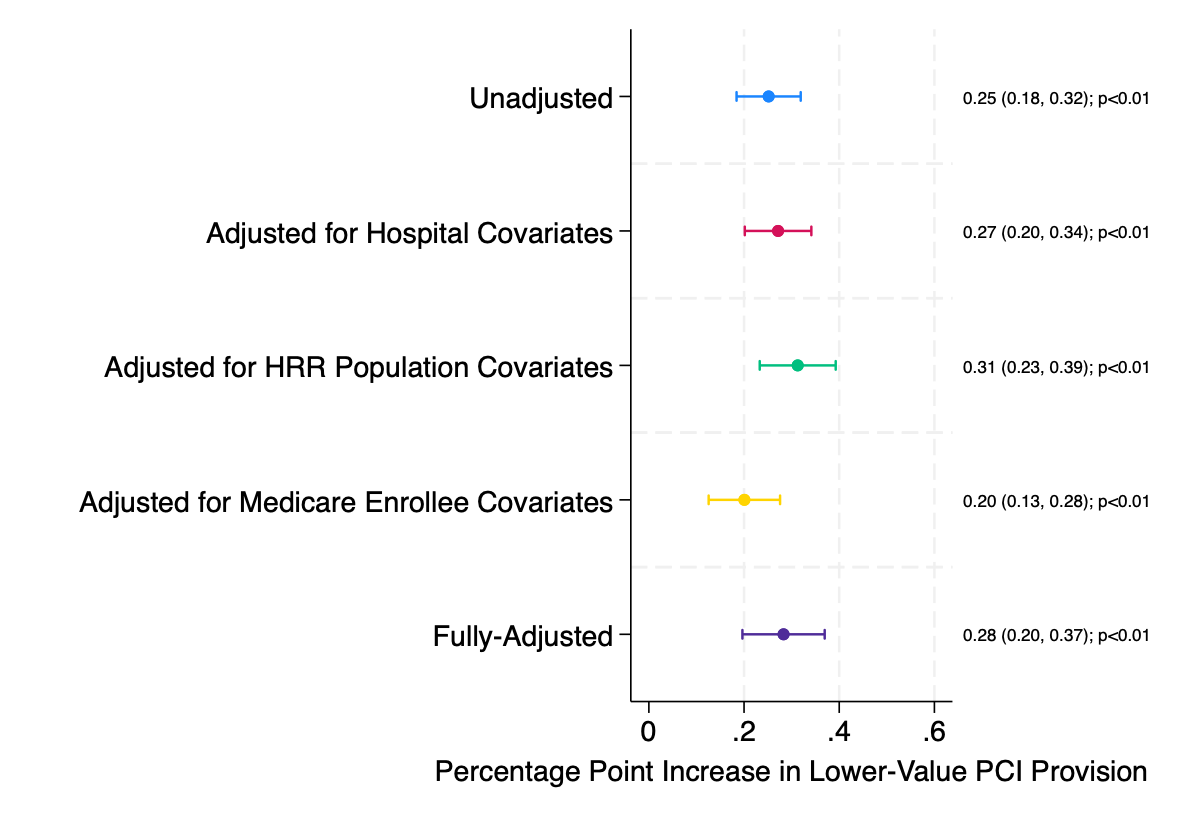

Supplement: S7 Fig — Note: PCI Provider Density is defined by PCI providers per 1,000,000 HRR population. Percentage point increase in Lower-Value PCI Provision Rate is per a unit increase in providers per 1,000,000. See S1 Appendix for CPT codes used to define PCI providers. HRR population covariates include the percentage of the HRR population aged 40–64 years, percent 65–74 years, and percent 75 + years; the percent of the non-elderly population that is uninsured; the percent of the adult population (18 years and older) with a physician diagnosis of coronary heart disease; and the median household income (inflation adjusted in 2021 dollars). Medicare enrollee covariates include the average age, the percent male, the percent dual eligible, and the average Hierarchical Condition Code score (a measure of ill-health) of Medicare FFS beneficiaries residing in the HRR where the hospital is located, as well as indicator of Medicare Advantage Penetration. Medicare Advantage enrollee characteristics include average age, percent male, and the percent dual eligible in the state in which the hospital is located. Hospital covariates include size (i.e., the number of hospital beds), safety-net hospital status (yes/no), urban/rural indicator, academic medical center status (yes/no), for-profit ownership (yes/no), and total hospital capital assets ($), as well as the quartile of hospital market concentration at the county level. Fully-adjusted models include all covariates. Regressions are Multilevel Linear Regressions that account for potential clustering within HRRs. Total hospitals in analyses that were unadjusted = 1,580; adjusted for hospital characteristics only = 1,570; adjusted for HRR population characteristics = 1,560; adjusted for Medicare FFS population characteristics = 1,580; in fully adjusted analyses = 1,550. (TIF) [file pone.0352150.s009.tif]

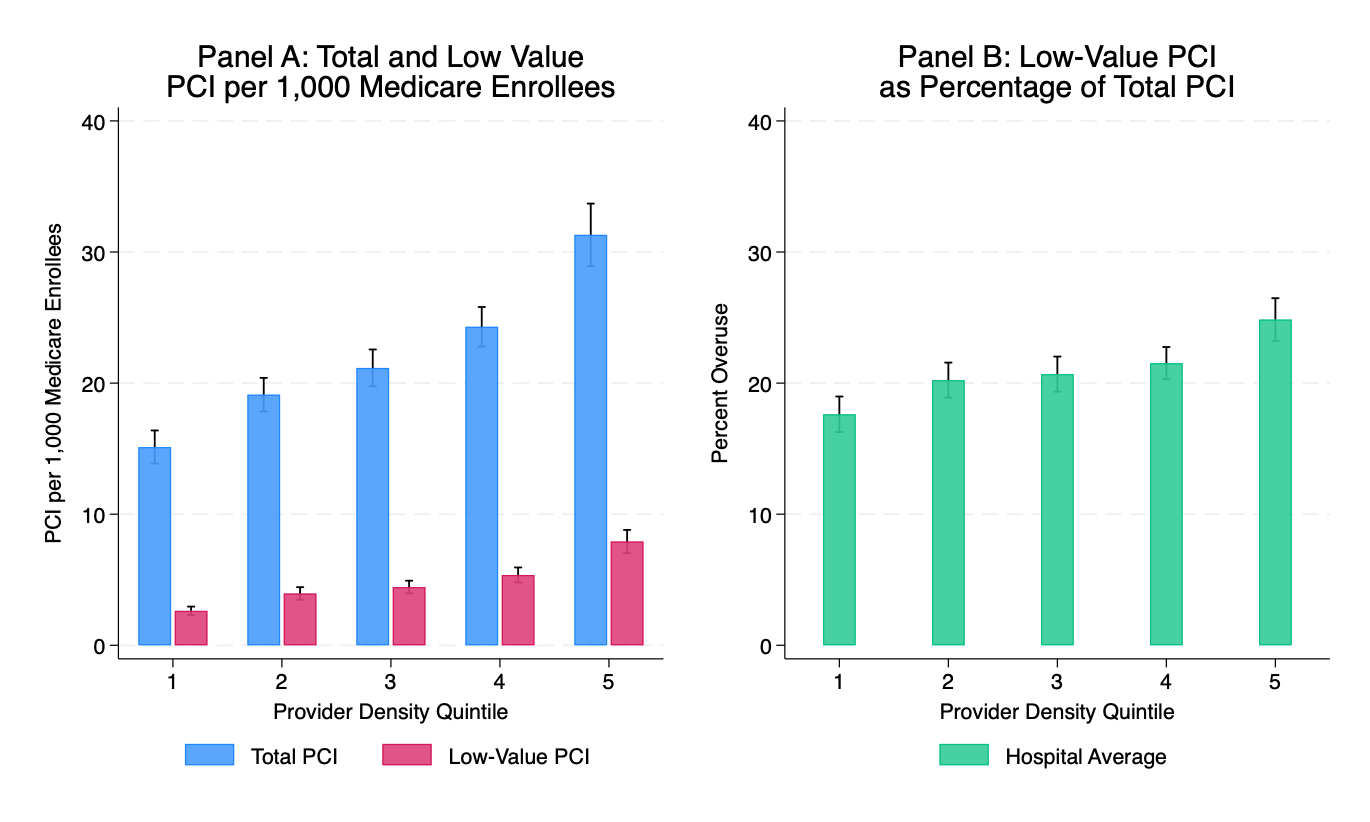

Supplement: S8 Fig — Note: PCI-Provider Density = PCI providers per 1,000,000 population, with HRRs divided into quintiles with approximately equal numbers of HRRs by this indicator for analyses in this figure. PCI utilization is per 1,000 Medicare enrollees in the HRR. Note that for PCI utilization outcomes only (Panel A), we included an additional n = 235 hospitals with < 10 PCIs in the study period. The Lower-Value PCI Provision Rate rate (Panel B) is calculated as the weighted average of percent overuse among hospitals within each HRR, using weights equal to total PCIs provided by each hospital during the study period (n = 1,580). (TIF) [file pone.0352150.s010.tif]
